# Improved Thermophysical Properties and Energy Efficiency of Aqueous Ionic Liquid/MXene Nanofluid in a Hybrid PV/T Solar System

**DOI:** 10.3390/nano10071372

**Published:** 2020-07-14

**Authors:** Likhan Das, Khairul Habib, R. Saidur, Navid Aslfattahi, Syed Mohd Yahya, Fazlay Rubbi

**Affiliations:** 1 Department of Mechanical Engineering, Universiti Teknologi PETRONAS, Bandar Seri Iskandar, Perak Darul Ridzuan 32610, Malaysia; likhan.das11@gmail.com (L.D.); mdfrs22@gmail.com (F.R.); 2 Research Centre for Nanomaterials and Energy Technology (RCNMET), School of Science and Technology, Sunway University, Petaling Jaya 47500, Malaysia; saidur@sunway.edu.my; 3 Department of Engineering, Lancaster University, Lancaster LA1 4YW, UK; 4 Department of Mechanical Engineering, Faculty of Engineering, University of Malaya, Kuala Lumpur 50603, Malaysia; navid.fth87@yahoo.com; 5 Sustainable Energy and Acoustics Research Lab, Mechanical Engineering Department, Aligarh Muslim University, Aligarh 202002, India; smyahya@zhcet.ac.in

**Keywords:** MXene, ionic liquid, nanofluids, thermophysical properties, PV/T system

## Abstract

In recent years, solar energy technologies have developed an emerging edge. The incessant research to develop a power source alternative to fossil fuel because of its scarcity and detrimental effects on the environment is the main driving force. In addition, nanofluids have gained immense interest as superior heat transfer fluid in solar technologies for the last decades. In this research, a binary solution of ionic liquid (IL) + water based ionanofluids is formulated successfully with two dimensional MXene (Ti_3_C_2_) nano additives at three distinct concentrations of 0.05, 0.10, and 0.20 wt % and the optimum concentration is used to check the performance of a hybrid solar PV/T system. The layered structure of MXene and high absorbance of prepared nanofluids have been perceived by SEM and UV–vis respectively. Rheometer and DSC are used to assess the viscosity and heat capacity respectively while transient hot wire technique is engaged for thermal conductivity measurement. A maximum improvement of 47% in thermal conductivity is observed for 0.20 wt % loading of MXene. Furthermore, the viscosity is found to rise insignificantly with addition of Ti_3_C_2_ by different concentrations. Conversely, viscosity decreases substantially as the temperature increases from 20 °C to 60 °C. However, based on their thermophysical properties, 0.20 wt % is found to be the optimum concentration. A comparative analysis in terms of heat transfer performance with three different nanofluids in PV/T system shows that, IL+ water/MXene ionanofluid exhibits highest thermal, electrical, and overall heat transfer efficiency compared to water/alumina, palm oil/MXene, and water alone. Maximum electrical efficiency and thermal efficiency are recorded as 13.95% and 81.15% respectively using IL + water/MXene, besides that, heat transfer coefficients are also noticed to increase by 12.6% and 2% when compared to water/alumina and palm oil/MXene respectively. In conclusion, it can be demonstrated that MXene dispersed ionanofluid might be great a prospect in the field of heat transfer applications since they can augment the heat transfer rate considerably which improves system efficiency.

## 1. Introduction

Since the industrial revolution, the demand for energy has been increasing day by day, thus it is anticipated that global energy demand will grow up to 30% by the year 2040 as stated in the *World Energy Report, 2019* by the International Energy Agency, (IEA) [1,2]. To meet this growing energy demand, fossil fuels are being brunt to a large extent, accounting for 87% of total energy supply. On this basis, the need for renewable energy resources is sought after due to the massive consumption of fossil fuels. Moreover, burning of these hydrocarbon deposits has a detrimental effect on the environment and global climate. Therefore, scientists are enforcing their efforts on proper utilization of green and renewable sources of energy mostly on solar, wind, tides, and geothermal energy [3]. Solar energy is considered the best alternative to supplement the consumption of fossil fuels in fulfilling energy demand. Furthermore, it is the most compelling source of renewable energy which can be utilized through numerous and ever-growing solar energy technologies—including solar thermal collectors, artificial photosynthesis, photovoltaics, solar ponds, and concentrating solar power plants [4,5,6]. Hybrid PV/T is a solar cogeneration approach that utilizes solar radiation and heat, producing both electrical and thermal energy. PV/T systems have gained immense popularity for residential and commercial applications because of their greenness and eco-friendly operation. The effectiveness of PV/T systems varies with several considerations such as incident angle of solar light, material, design, and working fluids [7,8]. Improving the thermal and optical properties of heat transfer fluid (HTF) is considered as the most convenient ways that augments the overall effectiveness of PV/T system. Water, thermal oil, ethylene glycol (EG), and ionic liquid are being employed as conventional working fluids in a hybrid solar PV/T system. Inclusion of solid nanoparticles into the heat transfer fluids can substantially improve optical and thermophysical properties [9,10,11]. The inclusion of nano-sized solid particles into base fluids, referred to as ‘nanofluids’, significantly alters thermophysical properties of the base liquids especially thermal conductivity [12,13,14,15,16]. Choi and Eastman [17] were the pioneers who coined the term “nanofluid”. Nanofluids have gained plenty of interest in recent decades as they have superior heat transport properties and also hold astounding possibilities in many clean engineering applications [18,19,20]; therefore, it is deemed to be the HTF for the next generation [21]. To date, researchers have conducted numerous investigations to improve the thermophysical properties of conventional HTFs by dispersing distinct nano additives such as metals, their carbides, oxides, or carbon nanotubes (CNTs) [22,23,24]. Recently, carbon-based nanoparticles and two-dimensional nanostructures have captivated ample attention due to their comparatively higher thermal conductivity, greater specific surface area, better compatibility with base fluid, better lubricating property, and ample availability when compared with other nanoparticles [25,26]. A broad number of studies were performed with graphite [27], carbon nanotubes(CNTs) [28,29], graphene [30], graphene oxide (GO) [31], nano diamond [32], and graphite flake [33] nanoparticles so far. Yu et al. [34] experimentally measured the thermal conductivity of EG based nanofluid with GO nanosheets where thermal conductivity was noticeably higher by 61% compared to pure EG, while the nanosheet concentration was 5 vol %. In another experimental work by Baby and Ramaprabhu [35], thermal conductivity of EG/graphene and water/graphene nanofluids were measured by transient hot wire method. Based on their observation, maximum improvement was about 64% at 50 °C when 0.056 vol % of functionalized 2D graphene was dispersed into deionized water. In another investigation, Wang et al. [36] experimentally inspected the influence of 2D graphene nanoparticles on thermophysical properties of ionic liquid [HMIM]BF_4_ and demonstrated that 18.6% improvement in the thermal conductivity was attained at 65 °C with nanoparticle loading as low as of 0.06 wt % while no significant decrease in specific heat was observed. Unpredictably, viscosity of synthesized nanofluids was found to be lowered than base fluids which could be ascribed to the self-lubricating property of graphene. Apart from this, an opulent number of studies were also carried out with imidazolium ionic liquid based (IL) nanofluids [37,38], concluding that imidazolium based ILs can be employed as an excellent base fluids due to their salient features like non volatility, high thermal stability, and good interaction with carbon-based nanoparticles [39]. Plentiful studies are found in the literature that find numerous nanofluids improve the heat transport efficiency of PV/T system [40,41,42]. Sardarabadi et al. [43] performed an experimental investigation to check the performance of a PV/T system using water/silica (SiO_2_) nanofluids with two different concentrations of 1 wt % and 3 wt %. The overall energy efficiency and thermal efficiency of the PV/T collector increased by 7.9% and 12.8% using nanofluid of 3 wt %, while for 1% they were found to have increased by 3.6% and 7.6% than pure water. In another study, phase change material (PCM) along with ZnO/water nanofluid were used in a solar PV/T module as coolant medium to see the simultaneous effect experimentally [44]. To accomplish this, they designed two systems: one with phase change material PCM, PCM/(PV/T); and PV/T only. PCM/nanofluid based PV/T system was subjected to 13% increment in electrical efficiency compared to a typical PV/T system, while an almost 9% augmentation in thermal efficiency was also noticed together with more than 23% overall exergy efficiency enhancement. Recently, Abdallah et al. [41] studied a PV/T system with MWCNT (multi-wall carbon nanotube)/water nanofluids to investigate the electrical and thermal efficiency. The findings demonstrated that adding nanoparticles into the water had prompted the enhancement of the thermophysical properties of the working fluid which further increased the overall efficiency of the system. For different particles concentration ratio varying from 0 to 0.3 vol % of MWCNT, the analyzed results reported that nanofluids with 0.075 vol % of MWCNT exhibited the best performances, reducing the panel temperature by 12 °C that led 83.26% overall system efficiency at maximum incident radiation. In addition, Al_2_O_3_/water, TiO_2_/water [45], Ag/water [42], sand-propylene glycol/water [46], and Fe_3_O_4_/water [47] were also explored to check their performance in PV/T systems in some studies. Reviewing the findings from literature, it can be attained that all relevant studies mainly focused on the optimization of solar PV/T systems using water-based nanofluid. Although overall thermal efficiency in some studies was satisfactory, they were only applicable at lower temperature ranges as water becomes thermally volatile at higher temperatures. Furthermore, variation in PV panel temperature and alteration of solar radiation were not studied in most cases. Therefore, it necessitates further studies with the aim of developing more promising nanofluids for solar PV/T system. MXene is a new family of 2D inorganic nanocomposite like graphene consisting of nano-sized layers of early transition metal carbides, carbonitrides, and nitrides [48]. MXene has a common formula of M*_n_*_+1_X*_n_*T*_x_* (*n* = 1, 2, and 3) which is synthesized from 3-D MAX phases [49] by selective etching of ‘A’ layer which is placed for a group IIIA or IVA element, M symbolizes early transition metal, T_x_ represents the surface termination (e.g., F, O, and OH) and X can be either carbon or nitrogen [50]. Since the surface termination/elemental composition is changeable [51], the properties of MXene can be altered rapidly, therefore rendering MXene more attractive than graphene. MXene has received immense research interest in numerous disciplines, such as nanomedicine, biosensors, electrochemical energy storage, and photothermal conversions [52,53,54]. In the present research, the authors formulate a novel ionanofluid with MXene and ionic liquid aqueous solution. The inclusion of 2D MXene into ionic liquid solutions has formulated a new class of ionanofluid. To the best of the authors’ knowledge, this is the first time MXene dispersed ionanofluid has been formulated successfully and its heat transfer behavior on a hybrid PV/T has been investigated. It will certainly be helpful for researchers and guide them in further investigations with this novel nanomaterial in heat transfer applications. Thermal conductivity, specific heat capacity, density, and assessments for this novel IL+ water/MXene are studied for the first time to the best of the authors’ knowledge. Thermal and electrical performance evaluation considering variable conditions (temperature, solar radiation) with this novel nanofluid in a hybrid PV/T solar system are new findings of this research work as well. Firstly, the authors formulate and characterize nanofluids and therefore, determine best concentration at which thermophysical properties are optimum. In consideration of their thermophysical properties, the authors favorably considered its possible application in hybrid PV/T system which could potentially serve as a more efficient alternative as heat transfer fluid for the application in hybrid PV/T solar system.

## 2. Materials, Methods, and Preparation

### 2.1. Materials

Ionic Liquid [MMIM][DMP] (1,3-Dimethylimidazolium dimethyl-phosphate) was supplied by Merck KGaA, Darmstadt, Germany. Table 1 represents the specifications of certificate analysis of [MMIM][DMP]. No further purification was accomplished during the sample preparation. Highly purified deionized water (Purity ≥ 99.998%) was prepared at nanomaterial research Centre of Sunway University, Malaysia.

### 2.2. Synthesis of MXene (Ti_3_C_2_)

In the synthesis of MXene (Ti_3_C_2_T*_x_*), the following materials were used without any further purification: MAX Phase material (Ti_3_AlC_2_) from Y-Carbon Ltd., Ammonium hydrogen difluoride (reagent grade 95%, Sigma Aldrich, Kuala Lumpur, Malaysia) and sodium hydroxide (97% purity, pellets, Sigma Aldrich) were obtained. Firstly, a solution of 2M and NH_4_HF_2_ was prepared precisely as the main part of the wet chemistry etching process. Afterwards, the dilution process of the ammonium hydrogen difluoride was conducted using DI water to produce 20 ml of solution, followed by magnet-stirring at 300 rpm for 1 h and at room temperature using hot plate magnet stirrer (RCT BASIC, IKA, Staufen, Germany). 1 g of Ti_3_AlC_2_ was weighed using microbalance (Explorer series, EX224, Ohaus, Parsippany, NJ, USA), then added to the uniform well-prepared NH_4_HF_2_ solution. Adding the MAX phase material to the prepared solution was performed slowly as the reaction is exothermic. The MAX phase suspension in the NH_4_HF_2_ was magnet-stirred at 300 rpm for 48 h and room temperature continuously to conduct the etching process. After the etching process, a dilute solution of NaOH was poured until the pH of the suspension reached 6 and was filtered and rinsed several times with deionized water. The washing process was conducted using an ultrahigh centrifuge (Sorvall LYNX 6000, Thermo Scientific, Waltham, MA, USA) four times (10 m each times) at 3500 rpm. The achieved multilayered MXene (m-Ti_3_C_2_) was then sonicated for 1 h by means of ultrasonic probe sonicator (‘FS-1200N’) to obtain delaminated flakes of the MXene (d-Ti_3_C_2_). The synthesized delaminated flakes of MXene nanomaterial was dried in a vacuum oven (VO 500, MEMMERT Germany, Schwabach, Germany) overnight.

### 2.3. Preparation of Ionanofluid

The formulation of ionanofluid samples was accomplished by dispersion of Ti_3_C_2_ nanoparticles into IL aqueous solution precisely. To achieve this, IL aqueous solution was prepared by dissolving 20 vol % of [MMIM][DMP] into 80 vol % of deionized water by magnetic stirring for 30 m at 45 °C and 700 rpm to obtain a homogenous mixture. The viscosity of the ionic liquid is high; therefore, adding water contributes to reducing the viscosity of the solutions. Furthermore, strong hydrophilicity of MXene will offer better stability with adding water [55]. Firstly, Ti_3_C_2_ nanosheet was precisely weighted using an analytical microbalance (Ohaus, Model: Ex224, accuracy: 0.0001 g, Parsippany, NJ, USA) and directly added into the IL solutions at three different concentrations of 0.05, 0.10, and 0.20 wt %. Next, all sample solutions were stirred using hot plate magnetic stirrer (RCT basic IKAMAG^®^ safety control, Staufen, Germany) for 1 h at 50 °C and 700 rpm. Finally, followed by an ultrasonic dispersion using a 1200 W, 20 kHz ultra sonicator (Ultrasonic Probe sonicator, Model: Fs-1200N, Hangzhou, China) for 30 m, a stable and well dispersed ionanofluid was formed by interrupting the hard agglomeration of MXene particles.

### 2.4. Characterization 

The surface morphology of Ti_3_C_2_ nanosheet was inspected with a SEM (scanning electron microscopy) “Tescan, Model: Vega, Brno, Czech Republic”. The SEM device was operated at 15 kV voltage and 10 mA current to obtain accurate and sharp images. In order to inspect the layered structure of Ti_3_C_2_, a sample area having significant number of layers was examined using SEM. Fourier transform infrared (FTIR) spectroscopy (Perkin Elmer^®^, Model: Spectrum Two^™^, Waltham, MA, USA) was employed to identify the chemical compositions and functional groups of the formulated nanofluids. The device was operated at a resolution of 4 cm^−1^ and each spectrum was obtained over 16 scans with a scan speed 0.2. The spectra were determined for infrared frequency region ranging from 400 to 4000 cm^−1^. Quantitative absorbance of all samples was measured as a function of wavelength using a UV–vis spectrometer (Perkin Elmer^®^, Model: Lambda 750, Waltham, MA, USA)

### 2.5. Thermophysical Properties Measurement

#### 2.5.1. Thermal Conductivity

**Figure 1:**
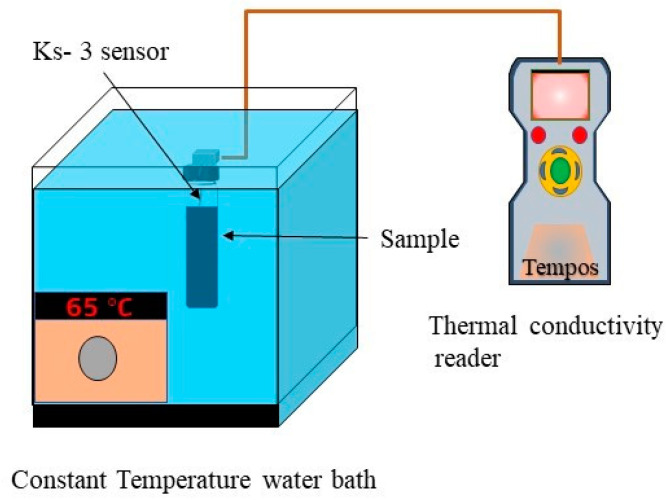
Setup used for measuring thermal conductivity.

To assess the thermal conductivity, a transient hot wire method operated thermal property analyzer (Model: Tempos, Pullman, WA, USA) was employed affording an accuracy of <±10% for each sample. A 6 cm, Ks-3 sensor based single heated needle was employed to detect the temperature by dipping the needle into the sample inside a hot water bath. Before measuring the thermal conductivity, the device was calibrated precisely using pure glycerin at 20 °C. The calibration details are given in Table 2 and an illustration of the used setup is shown in Figure 1. Before taking each reading, the samples were equilibrated for at least 10 min as temperature reaches the anticipated value. The precision of measurement was confirmed by repeating each measurement for 5 times and taking the mean value of iterations.

#### 2.5.2. Specific Heat Capacity

In this study, the specific heat capacity (*c*_p_) measurements of all samples were obtained using a (DSC). DSC-1000/C (Linseis, Germany), is a high resolution (0.03 µW) instrument and the measurements are conducted using an aluminum crucible of 40 µL. The temperature ranges from 26 °C to 60 °C with the heating rate of 10 °C/min. The synthesized samples are tightly sealed in a regular aluminum crucible with the capacity of 40 µL under a N_2_ ambiance with a flow rate of 20 ml/min. The repeatability and calorimetric precision of temperature are ±0.1 °C and ±1%, respectively. Temperature and enthalpy calibrations for DSC are carried out employing four standard reference samples (indium, tin, lead, and zinc) provided by the supplier. One uniform protocol for *c*_p_ measurements of the nanofluid samples are adjusted to validate the precision of results. However, the measurement uncertainty ranged from 0.2% to a maximum 0.8%.

#### 2.5.3. Measurement of Viscosity

A rheometer (Anton Paar, Model: MCR 92, Graz, Austria) was employed to measure the viscosity of the samples at a revolution rate of 100 rpm. Accuracy of the measurement was ±1.0% (in the range of −40 to 200 °C temperature) as stated by product specification. Viscosity of each sample was measured for the temperature range from 20 °C to 50 °C.

#### 2.5.4. Measurement of Density

A densitometer (Anton Paar, Density Meter, Model: DMA^TM^1001, Graz, Austria) was employed to measure the temperature depended density. The densitometer was calibrated with air and pure distilled water by performing air test and water test respectively. The density of the sample was taken as a function of temperature from 20 °C to 60 °C with an accuracy of 0.0001 g/cm^3^ and repeatability of at 0.00005 g/cm^3^.

#### 2.5.5. Measurement of Thermal Stability

The thermogravimetric analysis (TGA) was performed to measure the thermal stability of each sample, showing the weight decomposition as a function of temperature. The analysis was performed with thermogravimetric analyzer (Perkin Elmer^®^, Model: TGA 4000, Waltham, MA, USA). All the samples were heated from 30 °C to 500 °C in a compact ceramic furnace with a heating rate of 10 °C/min and a flow rate of 19.8 ml/min of N_2_ at 1.9 bar.

#### 2.5.6. Measurement of Zeta Potential

Zeta potential is a common technique to measure the stability of nanofluids and colloidal solutions [56]. To assess the zeta potential measurement of the prepared ionanofluids at different concentrations, a particle analyzer (Litesizer-500, Anton paar, Graz, Austria) was employed. The measurements were repeated for at least three times for each sample. 

### 2.6. Physical Model of PV/T System

In a hybrid PV/Thermal system for controlling the temperature rise of PV module various active and passive techniques were employed by researchers. Many of them used nanofluid as a coolant at the back of the PV panel to lower the temperature, especially in hot arid climate for better electrical efficiency. The specification of PV panel used for simulation purpose in PV/thermal system is listed in Table 3.

**Figure 2:**
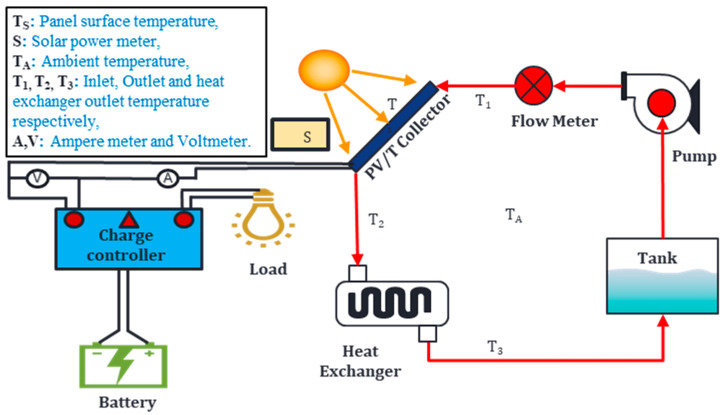
Typical model of a conventional PV/T system.

In this part, a numerical investigation is carried out to assess the performance of newly developed ionic liquid solution/MXene based nanofluid in PV/T system. The proposed work consists of hybrid PV/T system in which alumina/water, MXene/palm oil and IL+ water/MXene based nanofluid were employed and their performance are compared with water alone as a coolant numerically. A typical model of a PV/T system is presented in Figure 2, where the red color path indicates the thermal circuit and another one indicates the electrical circuit.

### 2.7. Numerical Modeling of PV/T Solar System

**Figure 3:**
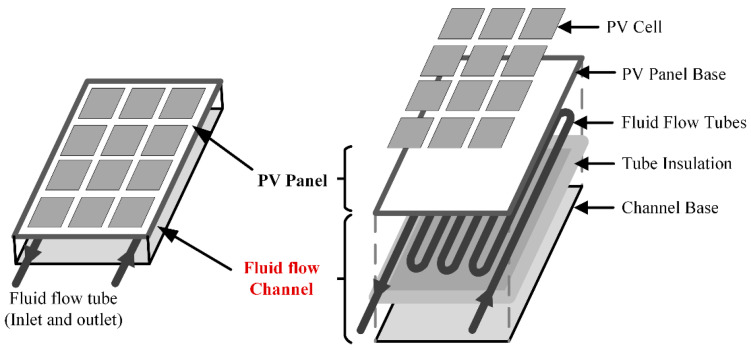
Schematic diagram of the proposed nanofluid backflow channel-based PV panel.

The problem under investigation comprises of PV module of 300 Watt which consist of four layers namely: PV solar cell, EVA (encapsulated vinyl acetate) on both sides of the solar cell, and a tedlar layer. Beneath the PV module, a heat exchanger in the form serpentine copper tubing is mounted (See Figure 3). The thicknesses of solar cell, EVA, and tedlar layers are 0.3 mm, 0.5 mm, and 0.1 mm respectively, remaining dimensions are the same as the PV panel i.e. (1955 mm × 982 mm). Finite element method-based Multiphysics software, COMSOL (Burlington, MA, USA) is used for numerical study. CFD and heat transfer modules of COMSOL are used to assess the performance parameters of PV/T system. It is assumed that the nanofluid flow is steady, three-dimensional, incompressible, and laminar. Transmissivity of EVA is about 100%, the role of dust on PV surface absorptivity is assumes negligible and temperature variation along the thickness of PV module assume to be zero. Furthermore, homogeneous mixture of nanoparticles in the base fluid is assumed (i.e., no particle agglomeration). In this study, [MMIM][DMP]+water/Ti_3_C_2_, palm oil/Ti_3_C_2_ based nanofluid with 0.2 wt % nanoparticle concentration are used as thermal conductivity is highest at this concentration. Thermal conductivity corresponds to 0.2 wt %, with different temperatures fitted to third order polynomial using regression analysis and inserted into a COMSOL environment using a user defined function. For alumina/water nanofluid, the Maxwell model is used for modelling thermal conductivity in which values of 0.611 W/m.K for water and 40 W/m.K for Al_2_O_3_ are used (see Equation (1)).

(1) $$k_{nf}=k_{bf}\frac{k_{s}+2k_{bf}-2\phi \left(k_{bf}+k_{s}\right)}{k_{s}+k_{bf}+2\phi \left(k_{bf}+k_{s}\right)}$$

For modelling temperature dependent viscosity of IL+ water/MXene and palm oil/MXene nanofluids, regression analysis of experimental data is performed. For IL+ water/Mxene nanofluid, thermophysical properties are experimentally obtained in present studies while previous experimental data has been used for palm oil/MXene nanofluids for same operation conditions [24]. The equation is incorporated into COMSOL using UDF (user defined function), as with thermal conductivity, and used for simulation purpose. For Al_2_O_3_-water nanofluid Brinkman model is used for modelling viscosity. For solid domain in the PV/T system heat conduction equation is used to account for heat transfer. Thermal transport from PV panel surface to flow channel is solved by a heat conduction equation as shown below in Equations (2)–(4).

(2) $$-\left(\frac{k}{\rho C_{p}}\right)\left(\frac{\partial^{2}T}{\partial x^{2}}+\frac{\partial^{2}T}{\partial y^{2}}+\frac{\partial^{2}T}{\partial z^{2}}\right)=\alpha_{p}G-E_{e}-h_{panel-ted}\left(T_{penal}-T_{ted}\right)$$

The equation shows heat transfer from the PV panel to tedlar layer. Similarly, other thermal energy equations between other layers can be written in a similar fashion. Here $\alpha_{p}$ is the absorptivity of the panel, *G* is the irradiance, *Ee* is the electrical energy output, and $h_{panel-ted}$ is the heat transfer coefficient between panel and tedlar layer. Similarly, other heat transfer coefficients between the layers are defined in Equations (3) and (4). Values of these are adopted as constant and are listed in Table 4 as thermal and optical properties of PV/T system. 

From the tedlar to serpentine tubing:

(3) $$-\left(\frac{k}{\rho C_{p}}\right)\left(\frac{\partial^{2}T}{\partial x^{2}}+\frac{\partial^{2}T}{\partial y^{2}}+\frac{\partial^{2}T}{\partial z^{2}}\right)=-h_{penal-ted}\left(T_{p}-T_{td}\right)-h_{ted-tubing}\left(T_{ted}-T_{tubing}\right)$$

From the serpentine tubing to nanofluid:

(4) $$-\left(\frac{k}{\rho C_{p}}\right)\left(\frac{\partial^{2}T}{\partial x^{2}}+\frac{\partial^{2}T}{\partial y^{2}}+\frac{\partial^{2}T}{\partial z^{2}}\right)=-h_{ted-tubing}\left(T_{ted}-T_{tubing}\right)-h_{tubing-nf}\left(T_{tubing}-T_{nf}\right)$$

The conjugate heat transfer equation is used for flow in the collector with both conduction and convection shown in Equation (5). Finally, the mass and momentum equation laminar fluid flow are given by Equations (6)–(9).

(5) $$\rho_{nf}C_{Pnf}\left(u\frac{\partial T}{\partial x}+v\frac{\partial T}{\partial y}+w\frac{\partial T}{\partial z}\right)=K_{nf}\left(\frac{\partial^{2}T}{\partial x^{2}}+\frac{\partial^{2}T}{\partial y^{2}}+\frac{\partial^{2}T}{\partial z^{2}}\right)$$

(6) $$\frac{\partial u}{\partial x}+\frac{\partial v}{\partial y}+\frac{\partial w}{\partial z}=0$$

X-momentum:

(7) $$\rho_{nf}\left(u\frac{\partial u}{\partial x}+v\frac{\partial u}{\partial y}+w\frac{\partial u}{\partial z}\right)=\frac{-\partial P}{\partial x}+{µ}_{nf}\left(\frac{\partial^{2}u}{\partial x^{2}}+\frac{\partial^{2}u}{\partial y^{2}}+\frac{\partial^{2}u}{\partial z^{2}}\right)$$

Y-momentum:

(8) $$\rho_{nf}\left(u\frac{\partial v}{\partial x}+v\frac{\partial v}{\partial y}+w\frac{\partial v}{\partial z}\right)=\frac{-\partial P}{\partial y}+{µ}_{nf}\left(\frac{\partial^{2}v}{\partial x^{2}}+\frac{\partial^{2}v}{\partial y^{2}}+\frac{\partial^{2}v}{\partial z^{2}}\right)$$

Z-momentum:

(9) $$\rho_{nf}\left(u\frac{\partial w}{\partial x}+v\frac{\partial w}{\partial y}+w\frac{\partial w}{\partial z}\right)=\frac{-\partial P}{\partial z}+{µ}_{nf}\left(\frac{\partial^{2}w}{\partial x^{2}}+\frac{\partial^{2}w}{\partial y^{2}}+\frac{\partial^{2}w}{\partial z^{2}}\right)$$

The density $\rho_{nf}$ and heat capacitance, $C_{Pnf}$ of nanofluid is assumed constant and their values were derived from empirical correlation available in archival literature [43] given below:

(10) $$\rho_{nf}=\left(1-\phi \right)\rho_{bf}+\phi \rho_{s}$$

(11) $$Cp_{nf}=\left(1-\phi \right){\left(C_{P}\right)}_{bf}+\phi {\left(C_{P}\right)}_{s}$$

Energy balance is applied across the hybrid PV/T system given in Equation (12), which consists of irradiance coming from the sun, radiation from the panel surface, convection between PV/T system and ambient, thermal energy generated and the electrical power output.

(12) $$G-P_{el}-P_{th}-{Q_{conv}}^{\prime }-{Q_{rad}}^{\prime }=0$$

Convective and radiative heat transfer from PV/T system are given by Equations (13) and (14) as follows. The convective heat transfer and radiative heat transfer from panel are calculated on the basis of Newton’s law of cooling and Stefan–Boltzmann law respectively.

(13) $$-n\cdot \left(-k\nabla T\right)=h_{total}\left(T_{surface}-T_{ambient}\right)$$

(14) $$-n\cdot \left(-k\nabla T\right)=\varepsilon \sigma \left(T_{surface}^{4}-T_{sky}^{4}\right)$$

where $h_{total}$ is the total heat transfer coefficient evaluated as $h_{total}={\left(h_{forced}^{3}+h_{natural}^{3}\right)}^{\frac{1}{3}}$. This includes the both effect natural convection and forced convection over the panel. Where forced and natural convection heat transfer coefficient [46] are calculated by Equations (15) and (16).

(15) $$h_{natural}=1.78{\left(T_{amb}+T_{surface}\right)}^{\frac{1}{3}}$$

(16) $$h_{forced}=2.8+3.0V_{wind}$$

Whereas the sky temperature is calculated using Swinbank relation [46] as $T_{sky}=0.037536T_{amb}^{4}+0.32T_{amb}$. In Equation (14) ε is the emissivity of panel and σ is the Stefan–Boltzmann constant.

The electrical power and thermal energy output are expressed by

(17) $$P_{el}=V_{oc}*I_{sc}*FF$$

(18) $$P_{th}=mC_{p}\left(T_{out}-T_{in}\right)$$

Electrical and thermal efficiency are obtained by Equations (19) and (20), respectively

(19) $$\eta_{el}=\frac{P_{el}}{G*A_{c}}$$

(20) $$\eta_{th}=\frac{P_{th}}{G*A_{c}}$$

### 2.8. Boundary Conditions

Appropriate boundary conditions were employed across the domain as per the physics of problem. Across the top and bottom layer of PV module the boundary condition applied is $-n\cdot q=h_{c}\left(T_{amb}-T_{s}\right)$. Where **n** is the surface normal and $T_{amb}$ and $T_{s}$ are the ambient temperature and surface temperature, respectively. For the fluid domain the inlet boundary condition is specified as velocity inlet along *x*-axis i.e., u = U_o_, v = 0, w = 0 and T = T_o_, for solid boundaries no-slip condition is used (u = v = w = 0), however at the outlet zero pressure boundary condition is used (*p* = 0). For solid–fluid interface heat flux continuity at the interface is used ${\left(\frac{\partial T_{s}}{\partial n}\right)}_{f}=\frac{k_{s}}{k_{f}}{\left(\frac{\partial T_{s}}{\partial n}\right)}_{s}$, adiabatic boundary condition is employed for the side surfaces of the system. Moreover, the lowermost plate of the hybrid solar PV/T system remains insulated.

### 2.9. Meshing and Grid Independence 

**Figure 4:**
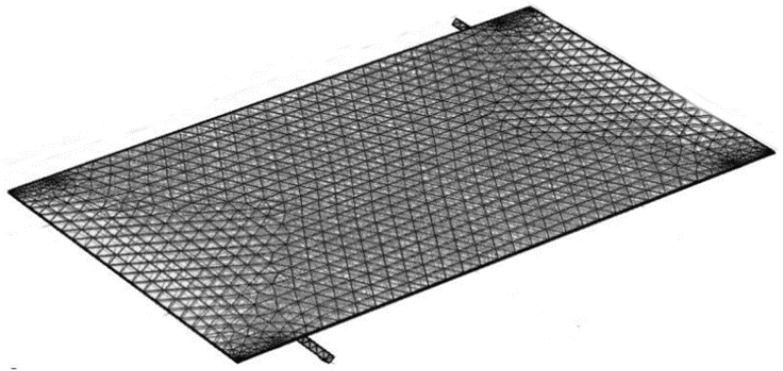
PV/T element with finite element meshing.

**Figure 5:**
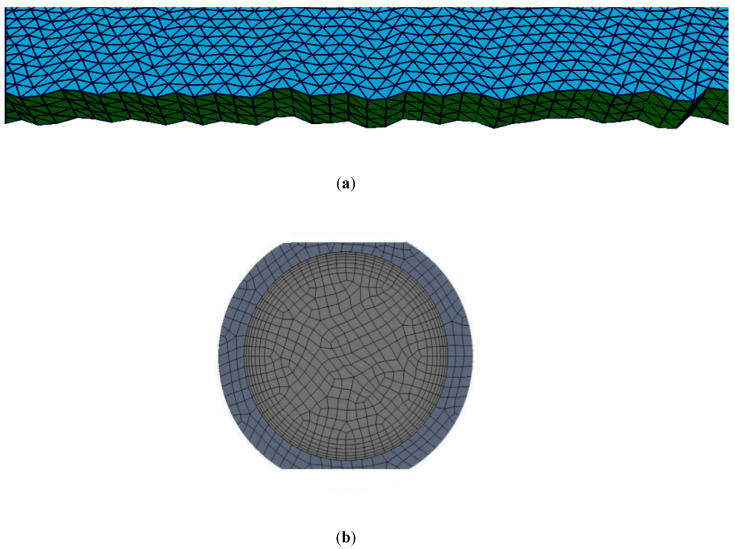
(**a**) Mesh along the thickness of panel. (**b**) Mesh inside and outside the tube.

The PV/T module was meshed in COMSOL Multiphysics® using the built-in physics-controlled mesh sequence setting shown in Figure 4. It consists of tetrahedral and triangular mesh element at the sub-domain and at the boundary respectively. The number of mesh elements increase at each boundary so that the heat transfer and flow fields can be resolved accurately. For grid independence, simulation at 1000 W/m^2^ and a mass flow rate of 0.05 kg/s is performed using water as a coolant with different mesh size (from coarser to finer) shown in Table 5. It was observed that there was no further change in panel temperature and outlet fluid temperature values after mesh no. 5. Thus, mesh no. 5 is chosen for simulation purposes. As far as quality measures are concerned, we checked the orthogonality, skewness, and growth rate of the elements and found satisfactory values of 0.65, 0.7, and 1.5 respectively. These values given us good quality mesh and the dialog box in COMSOL appears which states the minimum element quality to be 0.3319. If this value is <10^−4^ the quality is extremely poor. Apart from this there is one more parameter termed as aspect ratio which is 0.3, and it is a thumb rule in COMSOL if it is greater than 0.1, it would not affect the quality of the solution, in this simulation mesh curvature factor is 0.3 and mesh curvature cut-off is 0.001. In the boundary layer meshing, 8 layers with a stretching factor of 1.2 and scaling factor of 1 is used. The initial layer thickness is designated as 1/50 of the size of element on that boundary. The meshing along the thickness and around the tubing is shown in Figure 5a,b. The maximum and minimum deviation in the panel temperature for the six different meshes is 2.3% and 0.002% respectively. Similarly, for outlet fluid temperature, it is −2.94% and −0.22%. It was closely observed that, with consideration of mesh no. 6, the computational cost increases with meagre accuracy increment. Therefore, mesh no. 5 was considered for all the simulations.

## 3. Results and Discussions

### 3.1. Morphology and Characterization

**Figure 6:**
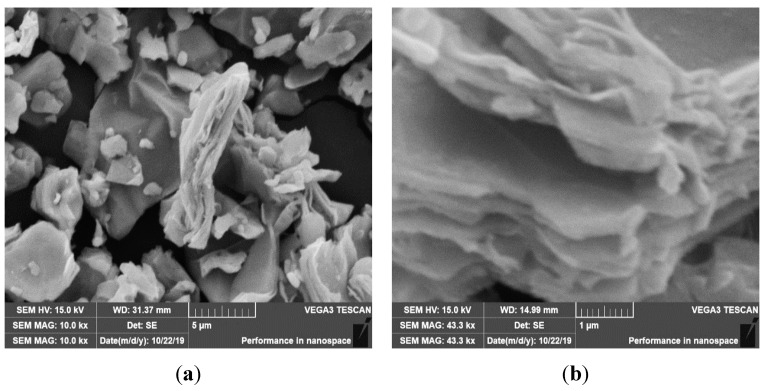
SEM micrographs of as synthesized Ti_3_C_2_ (MXene) (**a**) at 5 µm magnification (**b**) at 1 µm magnification.

Two-dimensional Ti_3_C_2_ was successfully synthesized from three-dimensional MAX (Ti_3_AlC_2_) phase which is evidenced by SEM micrographs presented in Figure 6a,b. The distinct self -staking layered structure of Ti_3_C_2_ which is clearly seen from SEM photographs is consistent with previous studies [57,58,59]. This layered structure is formed owing to the domino effect of van der Walls forces between two adjacent layers. Additionally, TEM images of multilayer flacks have a lateral size of up to 100 nanometers.

**Figure 7:**
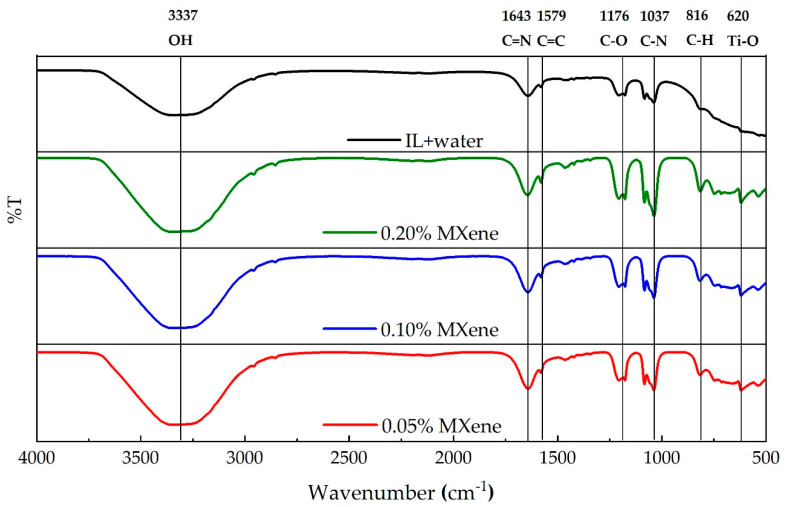
FTIR spectra of nanofluids at different concentrations.

The FTIR spectra of the formulated samples are presented in Figure 7. The broad peak appearing at wavelength 620 cm^−1^ is assigned to the chemically stable Ti–O bond, therefore, confirming the dispersion of Ti_3_C_2_ (MXene) in the IL aqueous solutions which is in good agreement with previous studies [60,61]. Additionally, a few more peaks appearing in the spectra of the sample solutions indicates the presence of stretching vibration bands of O–H bond at 3337 cm^−1^, C=N at 1643 cm^−1^, C=C at 1579 cm^−1^, C–O at 1176 cm^−1^, C–N at 1037 cm^−1^ and bending vibration band C–H at 816 cm^−1^. The assignment of these peaks strongly supports previous study in the literature for imidazolium-based ionic liquid [62]. The FTIR analysis implies that the dispersion of MXene into the IL solutions yields some functional groups which are stable, and no chemical reactions occur among the particles.

**Figure 8:**
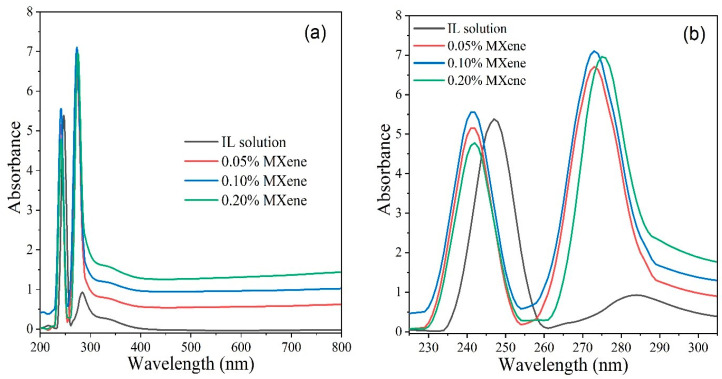
(**a**) UV–vis spectra of pure IL solution and nanofluids, (**b**) magnified view from 225 nm to 305 nm wavelength.

Figure 8a,b illustrates the UV–vis spectra of pure IL solution and Ti_3_C_2_ dispersed ionanofluids at different wavelengths. As shown in the figure, the absorbance peak appearing at wavelength of 291 nm increases substantially with the addition of Ti_3_C_2_ nanosheets, demonstrating that the optical absorption capability of the solution increases notably with the addition of two dimensional Ti_3_C_2_ nanosheet. Similar trends were observed from past studies with carbon-based 2D nanomaterials [34,63]. Moreover, the linear relationship between the nano additives concentration and absorbance justifies the Beer’s law fairly. This phenomenon is attributed to the high absorption ability of carbon and carbon-based nanocomposites. It must be noted that higher absorption ability of a fluid provides better performance for energy storage system specially for solar energy storage system [64].

Stability is substantially related to electro-kinetic potential differences between the stationary layer attached solid nanoparticles and dispersion medium [65]. A nanofluids must be stable enough before taking it for application. A nanofluid without stability will not be applicable even though it exhibits outstanding thermophysical and optical properties. The stability of [MMIM][DMP]+water/Ti_3_C_2_ nanofluids were measured in terms of zeta (ζ) potential. Table 6 illustrates the values of ζ potential measured as a function of temperature. As shown from the table, the prepared ionanofluids exhibit good stability without adding any surfactants (a commonly practiced chemical treatment to improve the stability by promoting interactions among particles). This is due to the fact that, MXene is strongly hydrophilic in nature that have strong interactions with water solution [66]. Furthermore, IL [MMIM][DMP] is also hydrophilic which also contributed to improving stability [67]. On the other hand, ζ potential of the sample shows a trend to increase with increasing concentration from 0.05 wt % to 0.10 wt % of Ti_3_C_2_ nanosheets. The increase in ζ potential can be explained by the fact that with increasing concentration the surface area of Ti_3_C_2_ nanosheet also increases, which in turn absorbs negative ions in their surface, therefore, the potential difference between the Ti_3_C_2_ surface and IL solutions increases. However, further increase in Ti_3_C_2_ concentration causes a decline in the ζ potential. A possible explanation for this is that although the surface area of the nanoparticles is increased, correspondingly the attractive forces between them are also increased causing their zeta potential to deteriorate. Similar trends were noticed in previous findings [68,69]. The effect of temperature on zeta potential value is also significant. For each case the ζ potential value increases significantly with increasing temperature which exhibits more stable structure with liquid particles. This is possibly due to the reduction of intermolecular forces between Ti_3_C_2_ particles as the Brownian motion increased with the temperature. Similar observations were also noticed for 2D graphene and carbon-based nanosheets in water solution [70]. As the ζ potential value increases with temperature, it clearly indicates the dispersion stability is improved at higher temperatures, which can be an advantage for better performance at higher temperatures. Taken together, these results suggest that a critical concentration is required to obtain the optimum value of the ζ potential which assures better stability of the suspension solution.

### 3.2. Thermophysical Properties

#### 3.2.1. Thermal Conductivity

**Figure 9:**
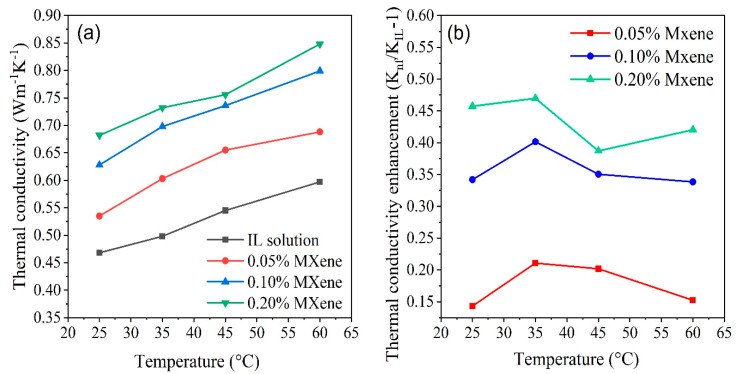
(**a**) Experimental values of thermal conductivity at different concentrations of Ti_3_C_2_ nanosheets for varying temperatures. (**b**) Thermal conductivity enhancement ratio at different temperatures with respect to IL+ water solution.

The thermal conductivities of the IL+ water solution and [MMIM][DMP]+water/Ti_3_C_2_ ionanofluids was determined at four distinct temperatures, ranging from 25 °C to 60 °C. The obtained values of each sample are depicted in Figure 9a and thermal conductivity enhancement of the ionanofluids (K_nf_/K_IL_-1) are shown in Figure 9b. As shown in Figure 9a, thermal conductivity of IL aqueous solution at 25 °C is 0.468W/m.K and rises up to 0.597 W/m.K as the temperature reaches 60 °C which completely agrees with the previous study at the same volume fraction and temperature of IL [71]. The trend of the thermal conductivity shows significant improvement with the addition of Ti_3_C_2_ nanosheets are into [MMIM][DMP]+water solution and continues improving as the loading of Ti_3_C_2_ nanosheets are increases. High surface area of nanoparticles, better heat transport properties, and good interaction with fluid nanoparticles are the primary facotrs that improve thermal conductivity. In addition, thermal conductivity of all samples increases remarkably with increasing temperature as a consequence of intensified Brownian motion of liquid molecules. As seen from Figure 9b, a maximum 47% enhancement in thermal conductivity occurs at 35 °C for 0.20% loading of Ti_3_C_2_ nanosheets, which is higher compared to 4.95% with graphene nanosheet/water nanofluids [72], 22.9% with graphene/[HMIM][BF_4_] ionanofluid [73], and 2% with GNP/EG nanofluids [74]. As higher thermal conductivity of HTFs offers higher thermal efficiency, it can be asserted that MXene dispersed nanofluid can be employed as novel HTF for solar PV/T systems.

#### 3.2.2. Specific Heat

**Figure 10:**
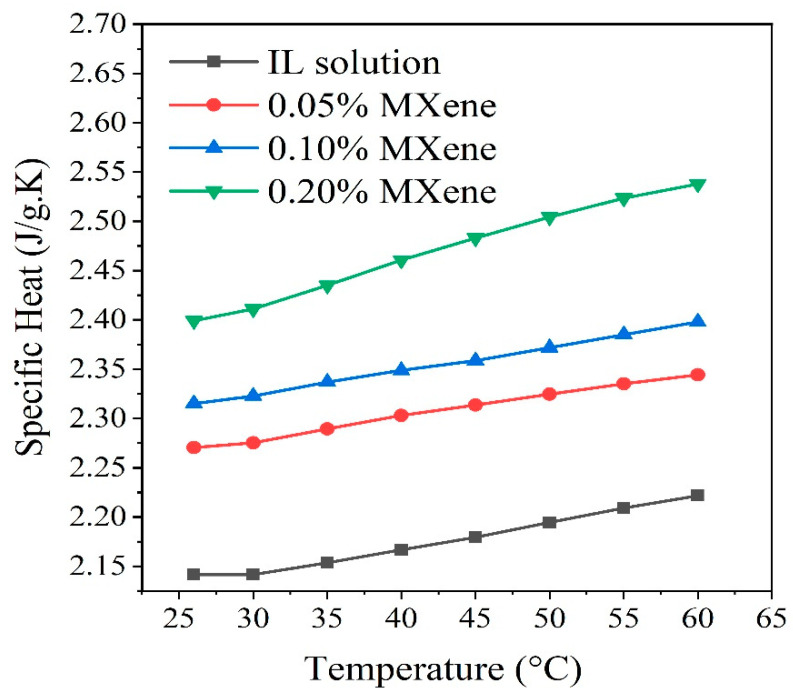
Specific heat capacity of IL aqueous solution and ionanofluids with various concentrations of Ti_3_C_2_.

The specific heat of [MMIM][DMP]+water solution and [MMIM][DMP]+water/Ti_3_C_2_ nanofluids for 0.05, 0.10, and 0.20 wt % of nanoparticle loading were measured in the temperature ranges from 26 °C to 60 °C. Figure 10 elucidates the variation of specific heat for base fluid and ionanofluids as a function of temperature. According to the trend lines, it is obvious that the dispersion of Ti_3_C_2_ nanosheets into the IL+ water solution causes the increase of specific heat of the solution which could be ascribed to the high specific surface energy of 2D Ti_3_C_2_ nanosheets. Similar trends were noticed from previous studies for trinary carbonate/CNT nanofluids [75], molten salt/SiO_2_ [76]. Furthermore, the specific heat is found to rise with increasing temperature for each sample. In other words, as the fluid heats up, the kinetic energy increases causing the fluid molecules to reach vibration state which enhances energy storage ability. Previous studies [36,77] showed similar trends which are consistent with the present study and mostly for imidazolium-based ionanofluids [78]. The specific heat of pure [MMIM][DMP]+water solution rises from 2.141 to 2.222 J/g.K with increasing temperature from 26 °C to 60 °C while for 0.05, 0.10, and 0.20 wt % of Ti_3_C_2_ ionanofluids, specific heat is noticed to increase linearly from 2.271 to 2.344 J/g.K, 2.315 to 2.397 J/g.K, and 2.399 to 2.537, J/g.K respectively for the same temperature enhancement. It must be noted that enhanced specific heat would result in a better outcome for solar thermal application, especially for energy storage systems.

#### 3.2.3. Viscosity

**Figure 11:**
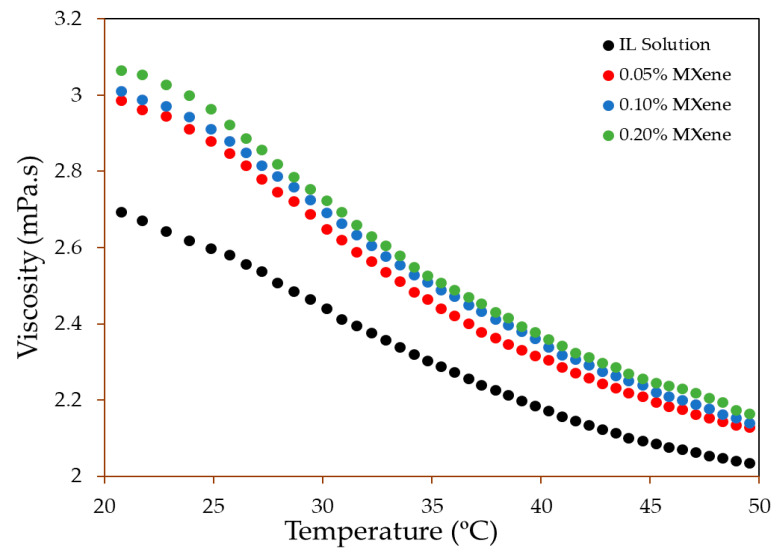
Dynamic viscosity of IL aqueous solution and ionanofluids with various concentration of Ti_3_C_2_.

Figure 11 illustrates the variation of dynamic viscosity of pure IL+water solutions and its nanofluids for different concentrations of Ti_3_C_2_ nanosheet in the temperature range from 25 °C to 60 °C. The viscosity of the pure IL+ water is lower than that of pure IL as less viscous water is added to IL [79]. It is obvious from the graph that the dynamic viscosity of the IL+ water solution increases from 2.694 mPa.s to 2.986 mPa.s when Ti_3_C_2_ nanosheets are added by 0.05 wt % while for 0.10 wt % and 0.20 wt % nanofluids, the viscosity further increases to 3.011 mPa.s and 3.066 mPa.s respectively. On the contrary, viscosity is noticed to decrease significantly as the temperature rises which complies with previous results [80,81]. The increase in viscosity of nanofluids attributes to the increasing shear stress with addition of Ti_3_C_2_ nanosheets into the IL+ water solutions. In other words, increasing the temperature weakens the intermolecular forces between the Ti_3_C_2_ particles and the solution itself. Hence, viscosity decreases as the temperature rises. However, lower viscosity is one of the major considerations for the application of HTF as higher viscosity increases pressure drop, therefore, it requires higher pumping power. In the current study, the increase in viscosity is almost negligible with increasing Ti_3_C_2_ nanosheet loading. Therefore, this conspicuous result emphasizes the reliability of using nanofluids in solar PV/T systems as a novel HTF.

#### 3.2.4. Density

**Figure 12:**
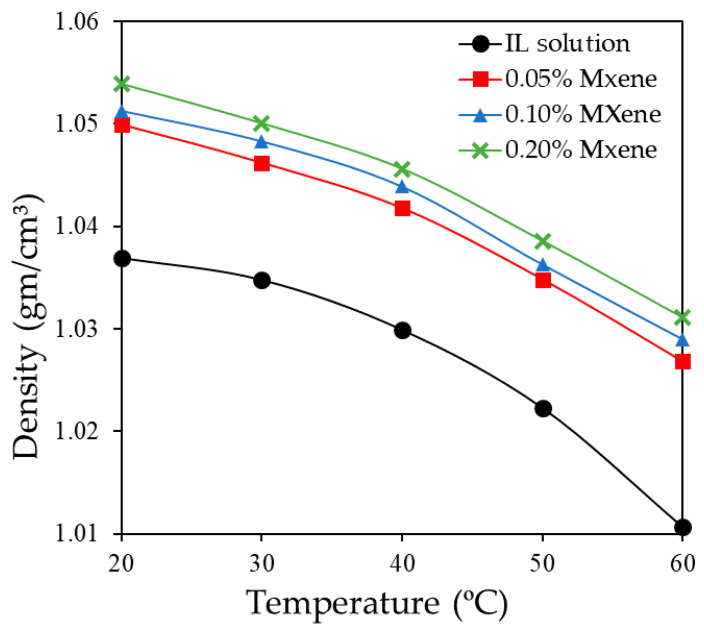
Density curve of IL+ water and IL + water/Ti_3_C_2_.

Density is also an important parameter for heat transfer systems as the pressure drop and pumping power significantly depend on density of the working fluids. Figure 12 illustrates the experimental density variation of the IL solution and ionanofluid at concentrations of 0.05, 0.10, and 0.20 wt % of Ti_3_C_2_ nanosheets. The result indicates a small increment of density when Ti_3_C_2_ nanosheets are dispersed into the IL solution at different concentrations which attributes to the addition of a high-density nanocomposites into the base fluid. In addition, the density decreases with increasing temperature as the kinetic energy of the fluids improves. In this study, maximum density occurs for the concentration of 0.20 wt % of Ti_3_C_2_ as expected as it rises from 1.037 gm/cm^3^ to 1.053 gm/cm^3^ at 20 °C. Since enhancement is lower than 2%, its effect the performance of the solar PV/T system will be negligible.

#### 3.2.5. Thermal Stability

**Figure 13:**
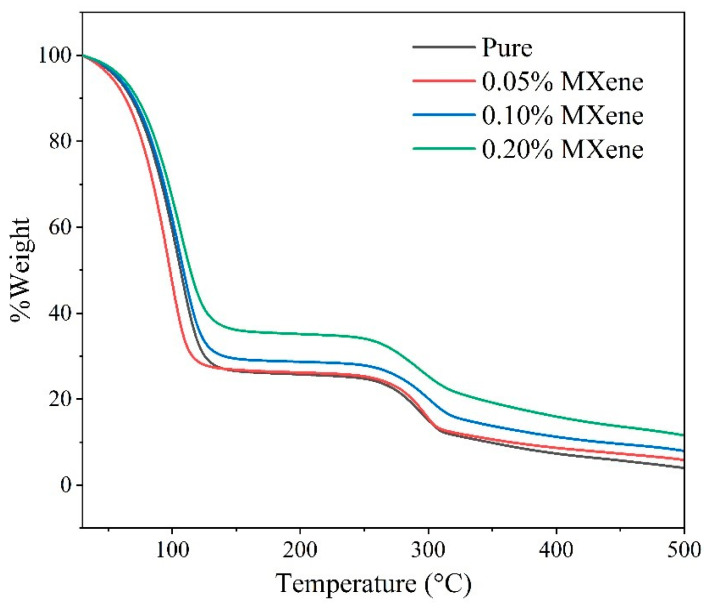
TGA curve of the IL+ water solution and ionanofluids at different concentrations of Ti_3_C_2_.

Thermal stability is another important parameter of thermal fluids since the applicability of thermal fluids greatly depended on their mass decomposition rate at working temperature [82]. The thermal stability is assessed by performing thermo gravimetric analysis (TGA) for each sample. Figure 13 illustrates the decomposition rate of the samples in terms of temperature. It is evident from the graph that all the samples are appreciably stable at up to 45 °C while initial decomposition of water starts at 45 °C for each sample. However, water contents are totally decomposed within 90 °C to 120 °C for different samples. It is obvious that the decomposition temperature of the nanofluids increases by dispersing Ti_3_C_2_ particles into the IL solution. This is attributed to the fact that some hydroxyl groups are introduced on the surface layers of the Ti_3_C_2_ particles that forms new functional groups containing high thermal stability. Furthermore, in the case of existing IL contents, no weight decomposes at up to 250 °C demonstrating all chemical bonds existing in the IL are strongly stable at up to 250 °C. Above 250 °C, all samples begin to decompose again and continue until all ionic liquids are fully decomposed nearly at 500 °C. The TGA curves demonstrate that the existence of Ti_3_C_2_ nanosheets in IL solutions does not lead to significant alteration in the decomposition rate of the IL solutions.

### 3.3. Validation of Numerical Model

A validation of model with previous results is depicted in Table 7. For validation purposes, a comparison is made with alumina-water nanofluid with numerical as well as experimental study of Sardarbadi et al. [83] and Lee et al. [84], respectively. It was observed that the results are in good agreement with previous results and shows an error of about 0.25% for panel temperature with numerical study and for electrical efficiency around 0.05% with experimental study.

### 3.4. Performance of Solar PV/T System

**Figure 14:**
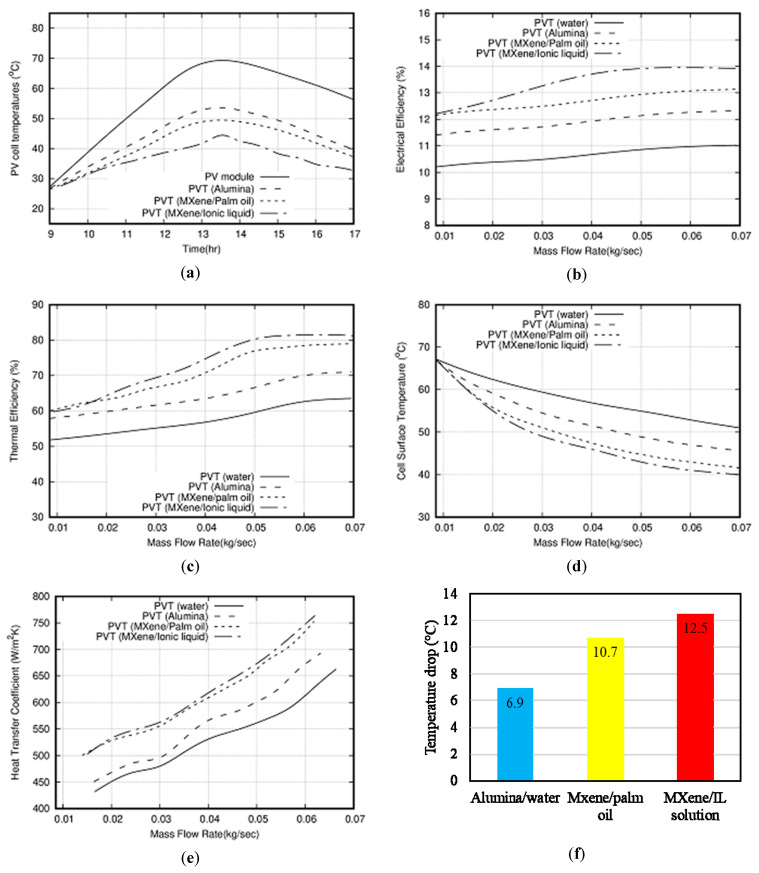
(**a**) Variation of PV panel temperature with time during a day for different types of coolant. (**b**) Electrical efficiency of PV/T system as a function of mass flow rate with different types of coolant. (**c**) Thermal efficiency of PV/T system as a function of mass flow rate with different types of coolant. (**d**) PV panel temperature as a function of mass flow rate using different types of coolant. (**e**) PV/T system heat transfer coefficient variation with mass flow rate using different types of coolant. (**f**) Drop in PV panel temperature by using different nanofluid types in comparison to a water-cooled system.

To maintain the PV module temperature in the permissible limit, three different coolants were examined in this study. All the simulations were carried out at 1000 W/m^2^ with nanoparticle concentration of 0.2 wt %. Figure 14a shows comparison with water, water/Al_2_O_3_, palm oil/MXene, and the newly developed IL+ water/MXene nanofluid to identify the effect on PV panel temperature during the daytime from 9:00 a.m. to 17:00 p.m. The simulation is scattered at at different times, alternatively we can say at different irradiance levels, and the average PV module temperature was noted for particular times or irradiances. Next, we plotted the curve for all times and joined them smoothly. It is not a transient situation. It was observed that MXene/IL+water solution shows a larger temperature drop across the PV panel, hence exhibiting better thermal performance. It is obvious from the figure that, at around 1:30 pm, the difference in the temperature between PV/T system using alumina nanofluid and the PV/T system using IL+water/MXene is 7.9 °C—i.e. IL+water/MXene nanofluids—showed a 15.19% increment in the heat removal performance relative to alumina based nanofluid and around 8.5% increment in the heat removal performance relative to palm oil/MXene nanofluid. This behavior is attributed to higher thermal conductivity of MXene nanoparticles as well as ionic liquid in comparison to palm oil as a base fluid.

Figure 14b presented the comparison of electrical efficiency with varying mass flow rate for three coolants as mentioned above. The electrical efficiency is enhanced with increasing flow rate, for instance, using IL+water/MXene and palm oil/MXene nanofluids, efficiency increases from 12.2% to 13.95% and 12.2% to 13.15% respectively when mass flow rate increases from 0.01 kg/s to 0.07 kg/s. Hence, by using palm oil based nanofluid in the hybrid PV/T system, a 7.8% electrical efficiency improvement is achieved in comparison to alumina nanofluid at 0.07 kg/s mass flow rate. Furthermore, by employing IL+water/MXene and at 0.07 kg/s mass flow rate, an electrical efficiency improvement of 14.4% is achieved in comparison to alumina/water nanofluid.

In Figure 14c, thermal efficiency variation with mass flow rate is depicted for different coolants. It is evident from the figure that higher mass flow rate offers better thermal efficiency of the PV/T system irrespective of type of coolant. At a maximum mass flow rate of 0.07 kg/s, water shows 63.3% thermal efficiency, meanwhile alumina/water shows 71.18% thermal efficiency, MXene/palm oil shows 79.13% thermal efficiency, and IL+ water/MXene shows 81.15% thermal efficiency. It is clear from the results that IL solution based nanofluid is performing better than that of palm oil and water based nanofluids and has high heat transfer capability. IL+water/MXene has increased the thermal efficiency by 14% in comparison to alumina/water nanofluid.

Figure 14d presents the PV panel temperature with mass flow rate. It is obvious from the graph that the initial range of mass flow rate is less than 0.015kg/s and there is not much variation in the PV surface temperature. However, a decreasing trend is observed with significant variation thereafter. This is due to the increased convection rate from the module which decreases the panel surface temperature with increases in mass flow rate. At a maximum flow rate of 0.07 kg/s, the temperatures of the PV surface with alumina/water, palm oil/MXene, and IL+ water/MXene nanofluids are 45.07, 42.5, and 40.5 °C respectively.

Finally, the heat transfer coefficient variation with mass flow rate is shown in Figure 14e. It was observed from the plot that heat transfer coefficient follows the increasing trend with mass flow rate irrespective of fluid used in the present study. Maximum percentage enhancement of 12.6% is achieved at 0.06 kg/s for IL+ water/MXene relative to the Al_2_O_3_/water nanofluid. Additionally, 2% enhancement is accomplished at 0.06 kg/s for IL +water/MXene relative to palm oil/MXene nanofluid. Figure 14f shows the temperature drop using three types of nanofluid in PV/T systems which is relative to water coolant PV/T system. The maximum temperature drop of 12.5 °C is achieved using IL+water/MXene as a coolant at maximum mass flow rate. It is clear from the results that IL+water/MXene is showing outstanding results in terms of thermal and electrical performance with its counterpart palm oil/MXene and alumina nanofluid.

## 4. Conclusions

In this present work, IL+ water nanofluids are formulated with novel 2D MXene (Ti_3_C_2_) nanosheet at three different concentrations (0.05, 0.10, and 0.20 wt %) and their thermophysical properties (thermal conductivity, viscosity specific heat, density, and TGA) and optical properties (SEM, FTIR, UV–vis) are measured. On the basis of their optical and thermophysical properties, authors were highly motivated to apply this synthesized nanofluids in solar cooling system. As a consequence, a simulation-based application is performed with synthesized ionanofluid along with two other nanofluids (water/alumina, olein palm oil/MXene) in a hybrid solar PV/T system to compare their thermal performances. The findings of the investigation can be concluded as follows:

- The 2D MXene were successfully synthesized from 3D MAX phase and SEM analyses were performed to inspect the morphology of MXene before formulating ionanofluids.
- The formulated ionanofluids showed good stability without adding any surfactants or chemical treatment. Optical property measurement also showed a significant improvement in absorbance (UV–vis analysis) capability which may be considered a promising aspect of solar energy storage systems. The FTIR analyses also showed that the MXene particles were well dispersed into the solution and they were chemically stable.
- Superior results were also obtained for thermophysical properties as the thermal conductivity enhancements are significant at each concentration of MXene; however, a maximum of 47% enhancement is noticed at 0.2 wt %. In addition, thermal conductivity increases substantially as the temperature rises from 20 °C to 60 °C.
- Interestingly, viscosity is found to be decreased by adding MXene nanosheets which might be attributed to their self-lubricating property. Specific heat increases with both increasing temperature and concentration while density is found to increase with concentration but decrease as temperature increases. TGA analysis also confirms that no significant decomposition occurs up to 60 °C within the samples.
- A simulation-based study has been conducted with IL+ water/MXene in a PV/T system along with two other nanofluids (water/alumina and palm oil/MXene) to assess the performance. IL+ water/MXene nanofluid with 20 wt % concentrations exhibits highest electrical efficiency, overall thermal efficiency, and heat transfer coefficient in comparison with water, Al_3_O_3_/water, and palm oil/MXene. Thermal efficiency of the considered PV/T system increases from 12.2 to 13.95% as flow rate increases from 0.01 to 0.07 kg/s with IL+ water/MXene. Moreover, thermal efficiency is also increased by 81.15% while, heat transfer coefficient is also increased by 12.6% and 2% for IL+ water/MXene compared to water/alumina and palm oil/MXene.

## Figures and Tables

**Table 1:** Properties of [MMIM][DMP].

Property	[MMIM][DMP]
Purity (HPLC)	≥98.0%
Identity (NMR)	passed
Density	1.27 g/cm^3^ (20 °C)
Water (KF)	≤0.1%
Halides (IC)	≤0.1%
Color	Yellow

**Table 2:** Calibration details of Tempos thermal conductivity analyzer.

Standard Sample	K_measured_	K_reference_ Wm^−1^k^−1^	Standard Deviation σk
Glycerin (20 °C)	0.289	0.282	±0.038

**Table 3:** Specification of PV panel.

Make and Model No.	Vikram Solar, ELDORA VSP.72.AAA.03
Material	Polycrystalline Silicon Cell
Dimension	1955 × 982 × 36 mm
No. of cells	72
Peak power	300 W
Maximum Voltage (Vmpp)	37.05 V
Maximum Current (Impp)	8.10 A
Open circuit voltage (Voc)	45.58 V
Short circuit current (Isc)	8.58 A
Weight of PV module	20.5 Kg
Operating temperature range	−40 °C to +85 °C
Standard test condition (STC)	1000 W/m^2^, AM 1.5, 25 °C

**Table 4:** Thermal and optical properties of PV/T system.

Properties	Values
Heat transfer coefficient from Panel to tedlar	150W/m^2^K
Heat transfer coefficient from tedlar to tubing	77 W/m^2^K
Heat transfer coefficient from tubing to nanofluid	66 W/m^2^K
Absorptivity of PV module	0.9
Absorptivity of tedlar sheet	0.5
Emissivity of PV panel	0.99
Thermal conductivity of EVA	0.311 W/m^-^K
Thermal conductivity solar panel	148 W/m^-^K
Thermal conductivity of tedlar	0.15 W/m^-^K
Thermal conductivity of thermal paste	1.9 W/m^-^K
Thermal conductivity of tubes	2700 W/m^-^K

**Table 5:** Grid independence test.

S.No.	Mesh Size (no. of Elements)	Panel Temperature (℃)	% Deviation	Outlet Temperature (℃)	% Deviation	Time of Solution (s)
1	2.5 × 10^5^	42.341	-	41.213	-	560
2	4 × 10^5^	43.872	1.2%	40.751	−1.13%	720
3	6 × 10^5^	44.003	0.29%	40.254	−1.23%	817
4	8 × 10^5^	44.118	0.26%	39.104	−2.94%	1115
5	1.5 × 10^6^	45.200	2.3%	38.889	−0.55%	1487
6	3.5 × 10^6^	45.201	0.002%	38.801	−0.22%	1815

**Table 6:** ζ-potential of ionanofluids different weight concentration Ti_3_C_2_.

Concentration	Zeta Potential (mV)					
	25 °C	Uncertainty (%)	45 °C	Uncertainty (%)	60 °C	Uncertainty (%)
0.05	−18.33	<5	−29.52	<5	−38.68	<5
0.10	−19.16	<5	−34.64	<5	−39.54	<5
0.20	−17.88	<5	−32.15	<5	−35.35	<5

**Table 7:** Code validation of PV panel surface temperature and electrical efficiency.

Panel temperature (°C)		Percentage Error	Remark
Present Research	Sardarabadi et al. [83]		
56.35	56.21	0.25%	At 1000 W/m^2^ and at a flow rate of 0.025kg/s (Numerical study of [83])
**Electrical efficiency**			
**Present research**	**Lee et al. [84]**		
12.15	12.22	0.5%	At 1000 W/m^2^ and at a flow rate of 0.05kg/s (Experimental study of [84])

## Nomenclature

**Nomenclature**		*σ*	Stefan Boltzmann Constant, W/(m^2^.K^4^)
*A_c_*	area of collector (m^2^)	*Subscripts*	
*c_p_*	Specific heat (J/K)	*amb*	ambient
*FF*	field factor	*el*	electrical
*G*	solar radiation intensity (W/m^2^)	*bf*	base fluid
*H*	convective heat transfer coefficient (W/m^2^. K)	*in*	inlet
*I_sc_*	short circuit current (A)	*out*	outlet
*k_nf_*	thermal conductivity of nanofluid (W/m.K)	*s*	solid particle
*k_bf_*	thermal conductivity of base fluid (W/m.K)	*th*	thermal
*k_s_*	thermal conductivity of nanoparticle (W/m.K)	*nf*	Nanofluids
*Q՛_conv_*	heat transfer due to convection (W)	*Abbreviations*	
*P_th_*	thermal power output (W)	FTIR	Fourier-transform infrared spectroscopy
*P_el_*	electrical power output (W)	NMR	nuclear magnetic resonance
*Q՛_rad_*	heat loss due to radiation (W)	IC	ion chromatographic
*T*	temperature (K)	KF	Karl Fischer
*V_oc_*	open circuit voltage (V)	IL	ionic liquid
*Greeks*		HPLC	high performance liquid chromatography
		TGA	thermogravimetric analysis
ζ	zeta potential, mV	SEM	scanning electron microscopy
*Φ*	nanoparticle weight fraction	PV/T	photovoltaic thermal
*ρ*	density, kg/m^3^	SEM	scanning electron microscopy
*η*	efficiency	UV–vis	ultraviolet–visible spectroscopy
*ε*	emissivity	UDF	user defined function
